# Study on Invadopodia Formation for Lung Carcinoma Invasion with a Microfluidic 3D Culture Device

**DOI:** 10.1371/journal.pone.0056448

**Published:** 2013-02-18

**Authors:** Shanshan Wang, Encheng Li, Yanghui Gao, Yan Wang, Zhe Guo, Jiarui He, Jianing Zhang, Zhancheng Gao, Qi Wang

**Affiliations:** 1 Graduate School of Dalian Medical University, Dalian, People's Republic of China; 2 Department of Biochemistry, Institute of Glycobiology, Dalian Medical University, Dalian, China; 3 Departments of Respiratory & Critical Care Medicine, Peking University People's Hospital, Beijing, China; 4 Department of Respiratory Medicine, The Second Hospital Affiliated to Dalian Medical University, Dalian, China; AMS Biotechnology, United Kingdom

## Abstract

Invadopodia or invasive feet, which are actin-rich membrane protrusions with matrix degradation activity formed by invasive cancer cells, are a key determinant in the malignant invasive progression of tumors and represent an important target for cancer therapies. In this work, we presented a microfluidic 3D culture device with continuous supplement of fresh media via a syringe pump. The device mimicked tumor microenvironment *in vivo* and could be used to assay invadopodia formation and to study the mechanism of human lung cancer invasion. With this device, we investigated the effects of epidermal growth factor (EGF) and matrix metalloproteinase (MMP) inhibitor, GM6001 on invadopodia formation by human non-small cell lung cancer cell line A549 in 3D matrix model. This device was composed of three units that were capable of achieving the assays on one control group and two experimental groups' cells, which were simultaneously pretreated with EGF or GM6001 in parallel. Immunofluorescence analysis of invadopodia formation and extracellular matrix degradation was conducted using confocal imaging system. We observed that EGF promoted invadopodia formation by A549 cells in 3D matrix and that GM6001 inhibited the process. These results demonstrated that epidermal growth factor receptor (EGFR) signaling played a significant role in invadopodia formation and related ECM degradation activity. Meanwhile, it was suggested that MMP inhibitor (GM6001) might be a powerful therapeutic agent targeting invadopodia formation in tumor invasion. This work clearly demonstrated that the microfluidic-based 3D culture device provided an applicable platform for elucidating the mechanism of cancer invasion and could be used in testing other anti-invasion agents.

## Introduction

Globally, lung cancer causes the most deaths in human beings among all cancers [1]. According to the World Health Organization, lung neoplasm is responsible for more than 1.3 billion deaths worldwide annually [2]. Recurrence and metastasis are the most major reasons of death in lung cancer patients despite advances in the treatment of primary tumors. The initial stage of cancer cell migration and invasion is the extension of cell protrusions in the direction of cell movement. The formation of these cell protrusions is usually driven by actin polymerization at the leading edge [3], [4]. During invasion and intravasation, the invasive cancer cells penetrate basement membranes using subcellular structures called invadopodia that localize matrix degrading activity to cell–substrate contact points [5], [6]. Therefore, the assay and investigation of invadopodia formation may provide more accurate insights in cancer invasion than other commonly used assays of cell-cell adhesion, and may be of great importance in cancer research in general. The molecular mechanisms of invadopodia formation in metastatic carcinoma cells are still unknown at present. Many reports on invadopodia formation with mammary adenocarcinoma, oral squamous carcinoma, colon cancer, melanoma, etc., have been published [5]–[13], whereas, only one involved lung adenocarcinoma [14].

Invadopodia are enriched with actin filaments, actin binding proteins, adhesion proteins, matrix proteinases and signaling proteins that regulate the actin cytoskeleton and membrane remodeling [15], [16]. The protrusive structure of actin filaments carries proteases that are able to degrade extracellular matrix (ECM) and are essential for metastasis [17], [18]. Factors involved in invadopodia formation include epidermal growth factor (EGF), matrix metalloproteases (MMPs), platelet-derived growth factor (PDGF), protein kinase C (PKC), neural Wiskott–Aldrich syndrome protein (N-WASP), and extracellular signal-regulated kinase (ERK), among which, EGF and MMPs are considered to be the most important parameters for this process. EGF induces dynamic cell protrusions associated with the actin cytoskeleton and EGF receptor activation stimulates signaling pathways that lead to enhancement of cell growth and cell motility [8]. Invadopodia formation induced by activation of EGF receptor signaling is considered to be an initial key step of cancer cell invasion and metastasis. In many different cancer cell types, the prognosis of a patient is inversely correlated with the overexpression and/or amplification of the EGF receptors [19]. Cancer cells with EGFR overexpression showed different responsiveness to EGF [8], [9]. MMPs belong to a family of 25 zinc-dependent endopeptidases that allow cells to both sense and remodel their environment through cleavage of extracellular factors and matrix proteins. They have been identified as important enzymes engaged by tumor cells during metastasis [20]. Recent data demonstrated that cells concentrated proteolytic activities on cell surface to help remove ECM barriers and facilitate cell migration. These activities were closely related to invadopodia [21], [22]. GM6001 (a broad range of MMP inhibitor) could inhibit the activities of MMPs. However, the functions of EGF and GM6001 on invadopodia formation in lung cancer invasion have not been studied yet.

Moreover, most of research on invadopodia so far was performed on two-dimensional (2D) surfaces with cells cultured on the glass slides coated with a thin layer of matrix. However, these experimental setups were far from being identical to the actual cellular environment [10]–[13]. Recently it has been shown that cells cultured in three-dimensional (3D) mode display gene expression profiles and biological activities that resemble the actual situation more closely than the cells cultured on 2D surfaces [23]. Since Basement Membrane Extract (BME) contains many ECM-like components, including laminin, collagen IV, entactin, and heparin sulfate proteoglycan [24], therefore, it can be used as a substitute of ECM in the 3D culture experiment for the purpose of simulating the tumor microenvironment.

Micro total analysis system, m-TAS, also called ‘lab-on-chip’ is a newly developed technology that has kindled increasing interest in biological and medical science by its virtue of reduced reagent and power consumption, less reaction time, portability for in situ use, low cost, versatility in design, and potentials for parallel operation and integration with other miniaturized devices [8], [25], [26]. This new technique also appears to provide some advantages for cellular biological analysis systems [27], [28], since the scale of the fluid inside a microchip matches the size of the cells. Besides, rapid and sensitive immunoassay systems for protein analyses with microfluidic systems, which are suitable for cell study, especially for 3D cell culture, have been demonstrated as well.

A microfluidic 3D matrix platform not only possesses the advantages of microfluidic chip but also more faithfully mimics the environment in vivo. It has been used effectively to reveal key differences in the morphology, metabolism and survival of normal and cancer cells. Current studies on the behavior of mammalian cells in microfluidic-based 3D matrix are emerging [29]–[35]. For example, Kamm **et al**., showed an excellent tumor cells invasion model in the investigation of the tumor-endothelial interactions with a microfluidic chip in 3D matrix [34]. These studies have confirmed that microfluidics is a useful measure by which cells are encapsulated in 3D matrix that simulates tissue architecture. The 3D matrix also makes it easier to observe the stretch of protrusions, overcoming the disadvantages of the 2D methods presently used in the invasion research, such as transwell [36], [37]. Therefore, the microfluidic 3D matrix platform may be an ideal carrier to build cancer invasion models. In the field of lung cancer research, however, few reports have been published to study the invadopodia formation in tumor invasion using the microfluidic 3D matrix platform.

In this work, we built a straightforward and practical microfluidic 3D culture device to assay the formation of invadopodia by human non-small cell lung cancer cell line A549. With this device, cells were cultured in 3D mode mimicking the situation in vivo. The mechanism of invadopodia formation and the effects of EGF and GM6001 in the process were tested and analyzed.

## Materials and Methods

### Design and fabrication of the microfluidic 3D culture device

**Figure 1:**
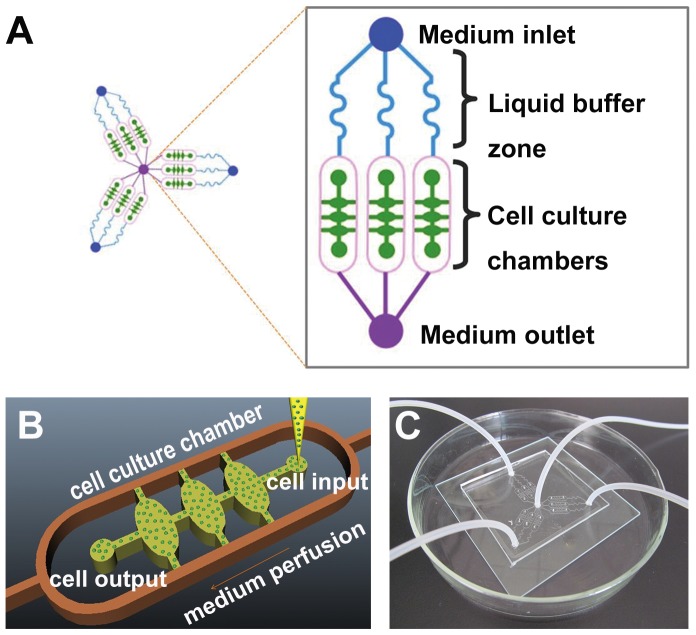
A microfluidic chip designed for the study of cancer cells invasion in 3D matrix. (A) Schematic representation of the microfluidic platform. Layout of the integrated microfluidic device is composed of three units sharing a common outlet, each of which contains an inlet, three parallel main channels, three cell culture chambers and an outlet. (B) A magnified illustration of one cell culture chamber. (C) Photograph of the microfluidic system.

The schematic representation of the device was shown in Figure 1A. The microfluidic 3D culture device was composed of three units sharing a common outlet through which the assay on three groups (one control group and two experimental groups) could be performed synchronously and in parallel. Each unit had an inlet for drug application or medium addition, three parallel main channels for liquid perfusion in liquid buffer zone, and three cell culture chambers. The liquid buffer zone was connected to the cell culture chambers by oval microchannels and the chambers were connected to the oval microchannels by two lateral channels for liquid spreading conversely. The size of all cell culture chambers was identical to each other (700 µm length, 400 µm width, and 0.4 µl volume). As shown in Figure 1B, the cell-BME mixture was seeded into the chambers through cell input and the excess mixture was effused from cell output.

The device was composed of a polydimethylsiloxane (PDMS, Sylgard Silicone elastomer 184, Dow Corning, Midland, MI, USA) layer and glass substrate. The PDMS layer was fabricated by replicate molding on the master, which was prepared by spin coating SU8-2035 negative photoresist (Microchem Corp, Newton, MA) onto a glass wafer and patterned by photolithography. Sylgard 184 PDMS base and curing agent were mixed thoroughly (10∶1 by mass), degassed under vacuum, and cast onto the master. The polymer curing process was carried out in an oven for 1 h at 80°C. After cooling, the PDMS layer was gently peeled off from the master and trimmed to size. Inlet and outlet holes were cored using a razor-sharp punch (Technical Innovations, Angleton, TX). After oxygen plasma treatment for 70 s, the piece of PDMS was bonded to a glass slide (1.0 mm thickness) irreversibly. The microfluidic device as shown in Figure 1C was sterilized with UV light for 30 min before being used.

### Cell culture and chamber filling with cell–BME mixture

Prior to cell culture, all components of the microfluidic system were autoclaved at 120°C for 30 min. Following cell manipulations were all performed in a sterile flow hood. Cells were incubated at 37°C in a standard 5% CO_2_ cell incubator with a humidified atmosphere of 95% air. The human non-small cell lung cancer cell line A549 was obtained from ATCC (American Type Culture Collection, Manassas, VA, USA) and cultured in F-12K medium (Sigma-Aldrich, St. Louis, MO, USA) supplemented with 10% fetal bovine serum (Gibco, Invitrogen, Inc, USA) and 100 U/ml penicillin and 100 U/ml streptomycin (Gibco, Invitrogen, Inc, USA). All experiments were carried out while the cells were in exponential growth phase. BME (R&D Systems, MN) was used as a substitute of ECM in the 3D culture experiment. To prepare cell encapsulation, BME was thawed at 4°C in a refrigerator overnight and A549 cells were then digested by trypsin (Gibco, Invitrogen, Inc, USA), centrifuged and resuspended at the density of 10^5^ cells/ml in ice-cold BME. Subsequently, cell–BME mixture was infused into cell culture chambers with a pipette to allow them to flow into each chamber. The mixture was stopped at the junction between the encapsulating buffer area and perfusion channels by capillary force. The cell-BME mixing and cell-loading process were performed on the ice to prevent BME from gelling. After filling the chambers with cell–BME mixture, the chip was kept at room temperature for 30 min to make the cell–BME mixture in a form of gel.

### Microfluidic device operation

This device was connected to syringe pumps via each inlet to drive the fluid flow. Drug solution and cell culture medium were simultaneously introduced into the microchannels at a fluid flow rate of 0.1 µL/min. The solutions diffused into cell-BME mixture across the oval microchannels. One syringe pump was connected to the common outlet to drain the waste solution produced by the device.

### 3D cell invasion assay

To evaluate the effects of EGF and GM6001 on the formation of invadopodia in A549 cell line, we divided the cells into one control group and two experimental groups (EGF group and GM6001/EGF group). In order to obtain an appropriate concentration for EGF and GM6001, we studied the effects of EGF and GM6001 on invadopodia formation with a concentration gradient microchip in a preliminary experiment and found that EGF and GM6001 at the concentration of 200 ng/ml and 10 µM, respectively showed the most significant influence on invadopodia formation (data was not shown). Therefore we used the two concentrations in our experiments. For the control group, the cells were cultured in the blank medium with continuous perfusion; for the EGF group, the cells were cultured in the medium containing EGF at 200 ng/ml concentration; and as for the GM6001/EGF group, GM6001 (Calbiochem, CA, USA) at the concentration of 10 µM was added to the medium before EGF perfusion similar to the EGF group. All cells in the three groups were supplied with the medium at the constant fluid flow rate of 0.1 µL/min for 12 h continuously.

### Immunofluorescence and morphology imaging of invadopodia

To assess invadopodia formation of the cells, we detected the expression of F-actin and cortactin by immunofluorescence assay. The cells were washed 3 times with PBS, and then fixed with 4% paraformaldehyde for 15 min at room temperature. The fixed cells were permeabilized in 0.5% Triton X-100 (AppliChem, Switzerland) for 15 min and rinsed and were then incubated with anti-cortactin antibody (mouse anti-human, abcam) diluted in 0.5% bovine serum albumin (BSA, Sigma) for 2 h in a moist chamber at 37°C. After washing 3 times with PBS, the cells were incubated with AlexaFluor 488-conjugated secondary antibody (donkey anti-mouse IgG, invitrogen, USA, 1∶800 dilution) and TRITC-phalloidin (Molecular Probes, sigma, Germany, 5 µg/ml) at 37°C in a moist cassette for 50 min. Then the cells were washed for another 3 times. Cells images were subsequently captured with confocal imaging system (confocal laser scanning microscope CLSM, Leica TCS SP5 II, Germany).

Furthermore, with the purpose of testifying actual invodopodia, we did the assay on invadopodia morphology of the three groups of cells in 3D with the confocal imaging system.

## Results

### Design of the microfluidic-based 3D culture device

**Figure 2:**
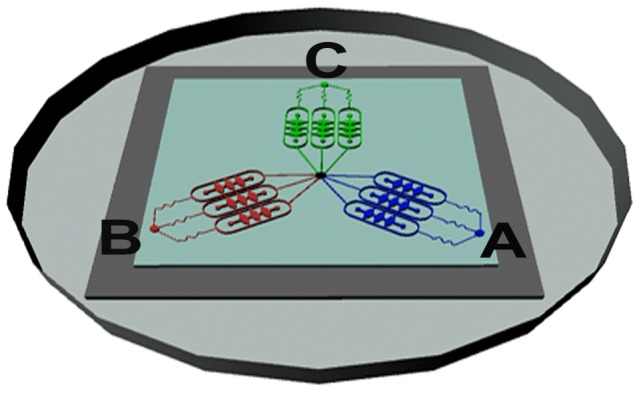
Illustration of medium flow direction in the microfluidic device. The blue, red and green indicators represented control group, EGF group, GM6001/EGF group respectively. These three indicators were perfused into microchannels from inlet A, B, C simultaneously and separately, while these indicators could spread out to cell chambers of both sides via oval microchannels uniformly and in parallel without crossing.

In this work, a microfluidic 3D cell culture device was designed and manufactured successfully to study lung cancer cell invasion (Figure 1C). For the view of validating the feasibility of the microfluidic platform for cell culture and fulfilling the parallel control test, indicators with different colors were injected into the microchannels of each group. As shown in Figure 2, the blue indicator represented control group, the red indicator represented EGF group, while the green indicator represented GM6001/EGF group. These indicators could spread in the cell chambers of both sides via oval microchannels uniformly and in parallel without crossing, showing that the microfluidic system was practicable for the assay of the invadopodium formation in A549 lung cancer cell line with several groups.

### Cell morphology and viability assay in 3D matrix

**Figure 3:**
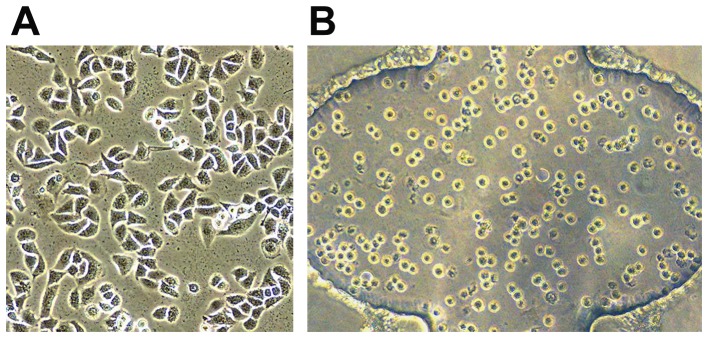
Morphology of A549 cells on 2D surfaces and in 3D matrix. (A) A549 cells were cultured on 2D surfaces. Cells exhibited to be flat with several protrusions. (B) A549 cells were cultured in 3D matrix. A majority of the cells were round without protrusions. Magnification: ×200.

In order to investigate the biological characteristics of cells in 3D cultures, A549 cells were embedded in BME. The morphological features of A549 cells in 2D and 3D cultures mode were compared. As shown in Figure 3, after 24 h culture, the cells appeared to be flat with several protrusions on 2-D mode (Figure 3A), whereas, their morphology was shifted to be spherical in 3D matrix (Figure 3B). This suggested that there was a significant difference in the shapes for the cells between the two culturing modes.

**Figure 4:**
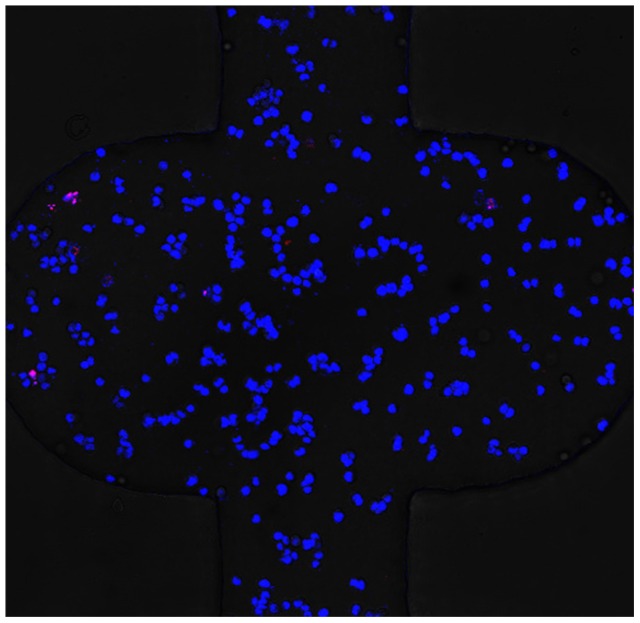
Fluoresent analysis of apoptotic in A549 cells. Cells were cultured on the 3D microfluidic device for 96 h and then stained by Hoechst and PI. Live cells were stained blue and dead cells were stained red. Magnification: ×200.

The cell apoptosis assays were performed using two specific fluorescence probes, Hoechst 33342 (Mbchem, China) and propidium iodide (PI, Molecular Probes, Eugene, OR, USA), as previously described [38] to evaluate the cell viability on the 3D microfluidic device. Stained nucleus were observed and photographed with live cells stained blue whereas apoptotic cells stained red. As shown in Figure 4, the cell apoptotic rate remained below 5% after 96 h culture. This result indicated that the survival period of A549 cells in gelled BME in our microdevice was long enough for the following studies. Also, the perfusion culture could provide an effective strategy to keep cells alive in 3D gelatinous matrix.

### Invadopodia formation assay for A549 cells invasion induced by EGF

**Figure 5:**
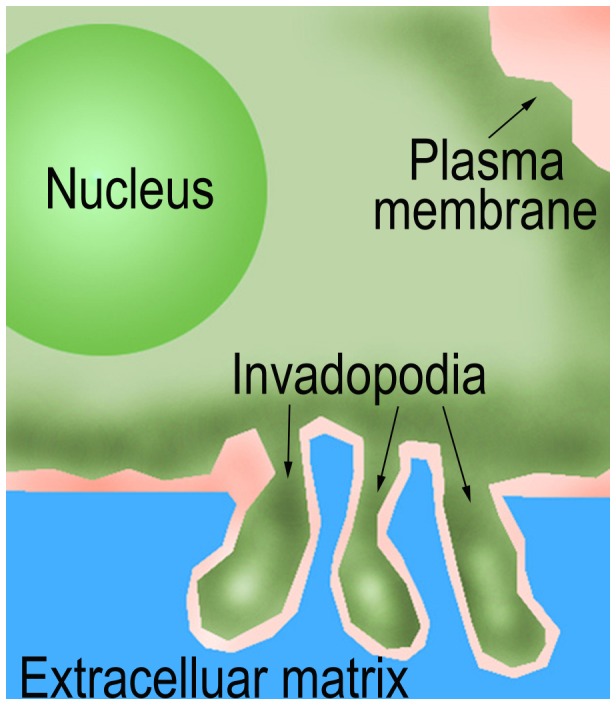
Scheme of invadopodia formation of cancer cells in 3D extracellular matrix in the microfluidic device.

**Figure 6:**
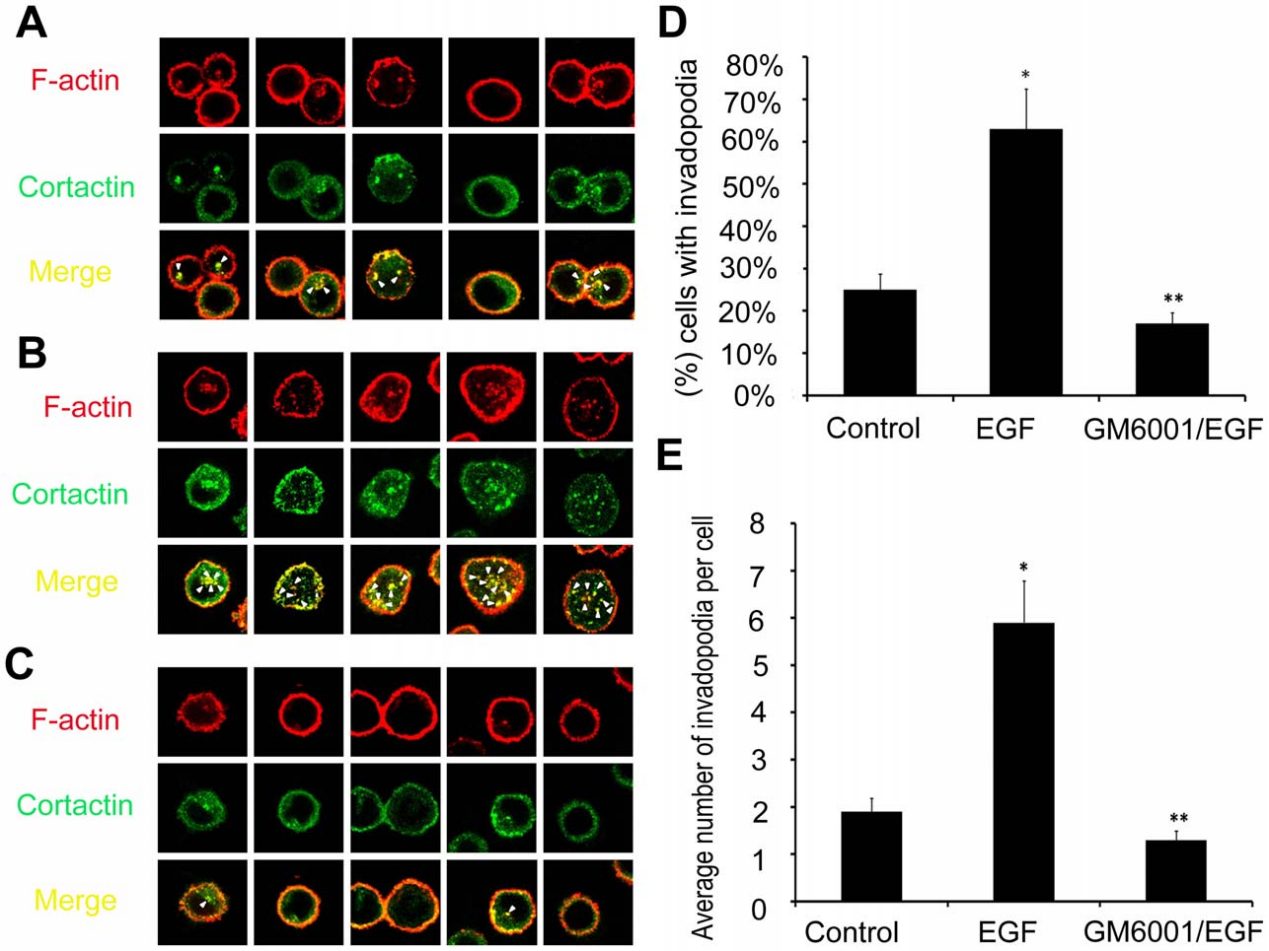
Invadopodia formation assay and quantification analysis with confocal system in A549 cells. The cells in control group (A), EGF group (B), and GM6001/EGF group (C), were cultured on a 3D microfluidic device for 12 h. The cells were stained green represented combined with cortactin, stained red represented combined with F-actin, and arrowheads in merge pictures indicated cells displaying invadopodia. (D) The percent of the cells with invadopodia formation. (E) The average number of invadopodia per cell. Error bars represented the SD of three different determinations. *Statistically significant between control group and EGF group; **statistically significant between EGF group and GM6001/EGF group, p<0.05. Magnification: ×1200.

**Figure 7:**
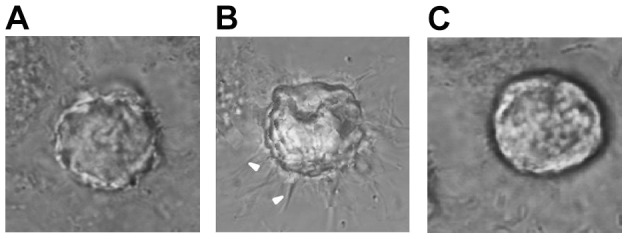
Actual invadopodia formation of A549 cells in control group (A), EGF group (B), and GM6001/EGF group (C) in 3D extracellular matrix in the microfluidic device with confocal system. Invadopodia could be obviously induced by EGF in (B), **while** this induction could be inhibited by GM6001 in (C). White arrowheads represented invadopodia. Magnification: ×1200.

Invadopodia formation is the major manner of cancer invasion and is associated with many proteins, including F-atin and cortactin that are considered as reliable indicators for cancer invasion. Therefore, in our study, we investigated the invasion of lung cancer through the assay of F-actin and cortactin to illustrate invadopodia formation in a 3D microfluidic matrix model. To investigate whether EGF was capable of inducing invadopodia formation for A549 cells cultured in our microfluidic device in 3D matrix, F-actin and cortactin expression were assayed in the three groups of cells. Figure 5 presented a model of invadopodia formation in the device and Figure 6 showed the expression of invadopodia protein of F-actin and cortactin assay by immunofluorescence. In the latter figure, F-actin expression was indicated in red color and cortactin expression in green, whereas merged images with yellow color suggested colocalization of the two proteins. The stimulation with EGF remarkably induced the formation of actin dot-like structures (Figure 6B) while approximately 64% of A549 cells showed evidence of invadopodia formation (Figure 6D) with an average of 5.8 invadopodia foci per cell, 3-fold greater than that of the control group (Figure 6E). This induction could be inhibited by GM6001 (Figure 6C). Furthermore, in order to demonstrate the actual matrix degradation and the morphology of invadopodia in cancer cells, we did the assay on invadopodia in 3D with confocal imaging system. As shown in Figure 7, similar to the assay by immunofluorescence, invadopodia could be evidently observed in the EGF group and this induction could also be inhibited by GM6001. These results indicated that EGF was an effective factor to induce invadopodia formation, a clear indication of invasion of lung cancer cells.

### GM6001 inhibition assay on invadopodia formation induced by EGF for A549 cells

In order to investigate whether the induction of invadopodia formation could be inhibited by MMP inhibitor (GM6001) in A549 cells, we performed the assay on the GM6001/EGF experimental group. As shown in Figure 6C–E, GM6001 neutralized the inductive effect of EGF and led to a decrease in the formation of actin dot-like structures to the level as low as that without EGF stimulation (the control group) or even lower. Only 17% of A549 cells formed actin dot-like structures (Figure 6D) and the cells displayed an average of 1.3 invadopodia foci per cell with the level near to that of the control group or even lower, suggesting that the EGF-induced formation of invadopodia could be blocked significantly by preincubation of cells with GM6001.

## Discussion

Compared with conventional platform, the microfluidic technologies boast unmatched advantages including high throughput, integration, miniaturization and parallelization, and may be used as a highly compact and integrated platforms, especially suitable for applications in biology, biochemistry, biotechnology, medicine, and in clinical studies [39]. More recently, a great number of studies for various cell-functional measurements based on this microfluidic system have been reported, and some of them equipped with micropumps to promote more efficient integration for biomedical field research [40], [41]. Most of these applications are involved with complicated microstructure, requiring extensive technical support, which, to some extent, limits the widespread use of this novel technique. Consequently, there is an urgent requirement to develop a simple, more maneuverable microfluidic system to accomplish complex cytobiology analysis.

It is important to mimic the environment surrounding the tumor cells in vivo to study tumor cells invasion and migration. Fortunately, 3D cell culture with matrix gel can satisfy this requirement. Compared to 2D cell culture mode, 3D cell culture mode has the following advantages. First, 3D culture system can provide the support or matrix similar to the growth microenvironment of cells in vivo that allows tumor cells to form a 3D structure, thus enabling researchers to establish the relationships between cells and cells or extracellular matrix through the cell-cell close connection and/or gap junction and connection [42]. Second, tumor cells cultured in 3D mode display morphology that resembles in-vivo. This is beneficial to researchers when observing and tracing the cell-cell adhesion and interaction, cell-matrix interaction and cell migration [34]. Third, cells in 3D culture mode have a significant difference from the 2D monolayer culture mode in gene expression, matrix secretion and cell function activity, etc [43].

In this work, we developed a simple but effective microfluidic device to investigate the role of EGF, GM6001 in invadopodia formation in the process of invasion for A549 lung cancer cell line in an in vivo-like 3D microenvironment. We built a microfluidic-based 3D matrix platform using BME as a substitute of ECM for 3D mode cell culture, and the chip was made by PDMS due to its excellent optical transparency and biocompatibility, and could provide a conducive environment for cell growth and proliferation. With this microfluidic device, invadopodia formation, which was regarded as a key determinant in the malignant invasive progression of tumors, can be simply assayed by the immunofluroescence of the biomarkers, such as F-actin and cortactin. Obviously, this assay is also more convenient and accurate than the commonly used methods such as traditional western blot and real-time PCR on other invasion parameters including cell-cell adhesion and interaction (E-cadherin and N-CAM loss), cell-matrix (ECM) adhesion and interaction, neoangiogenesis and lymphangiogenesis and so on for tumor invasion investigation [44]–[48]. To facilitate the study of cell behaviors in vitro, we applied an injection pump to support the cells with continuous supply of fresh automatically, thus maintaining cells in better condition in the device that mimics in vivo-like microenvironments. Since shear stress caused by fluid velocity is harmful for cell growth, so fluid velocity during perfusion of cell culture was relatively low and was dictated by the balance between adequate mass transport and limited hydrodynamic effects [49]. Compared with our previous simple chip [50], the operation on the new microfluidic chip became more practical, for example, the perfusion of the cells with medium and drugs could be through different ports (inlet A for control medium, inlets B and C for drugs), reducing the interference among different medium; also, the device kept three groups of cells under analysis exactly in the same condition in parallel before further culture. With this device, human lung cancer cell line A549 grew and propagated for at least 96 h. Multi-step operations from detection of cell viability to invadopodia-related protein analysis were achieved on a microchip continuously and conveniently, fully exploiting the integrated advantage of microfluidic technology over the conventional platform in terms of time and reagent consumption as well as sensitivity.

During the proliferation process of malignant tumor, migration through the ECM is a necessary step for cancer cells to invade adjacent tissues and metastasize to a distant organ. This process is dependent on the capacity of invasive cancer cells to breach the basement membrane. Invasive cells invade the surrounding environment by utilizing invadopodia which are actin-based membrane protrusions formed at contact sites between invasive tumour cells and the ECM with high ECM degrading capacity.

Behaviors of invadopodia are associated with cancer cell aggressiveness come together, including tyrosine kinase signaling such as EGF receptor signaling pathway, protease secretion and targeting, and cytoskeletal rearrangements for cell movements. EGF is a growth factor known to induce dynamic cell protrusions associated with actin cytoskeleton [8], [9], [51]. These protrusive structures carry proteases such as MMPs which comprise a highly regulated family of structurally related enzymes capable of degrading most, if not all, of the components of the ECM [52], [53], are able to degrade extracellular matrix [17], [18]. Thus MMP inhibitors which are able to protect tissue boundaries from being invading are considered as a potential therapy for anti-invasion.

In our study, A549 cancer cell invasion induced by EGF could be observed and quantified by measuring the percentage of the cells with invadopodia and the average number of invadopodia foci per cell. We provided evidence that epidermal growth factor receptor (EGFR) signaling played a significant role in invadopodia formation and related ECM degradation activity in A549 cells. Moreover, we found that invadopodia formation of A549 induced by EGF was inhibited by MMP inhibitor (GM6001). These results in lung cancer cells are consistant with the emerging evidences that suggest a critical role of EGF signaling pathway in the invadopodia formation as well as the invasiveness and metastasis of cancer cells [8], [9], [51], [54]. Since invadopodia are not vital for cell viability, it is suggested that anti-invadopodia therapy would be expected to have fewer side effects than current radio- and chemotherapy approaches in cancer therapeutics [11], [13]. Given that, further studies are required to understand invadopodia functions and to identify anti-invadopodia targets. With regard to the latter, invadopodia scaffold or signaling proteins may represent good targets for disruption of invadopodia formation without affecting important cellular processes that involve actin cytoskeletal rearrangements or membrane trafficking processes elsewhere in the cells. For instance, cortactin as a good candidate for anti-invadopodia therapy, plays an essential role in the formation of invadopodia but lacks of effect on cell viability [55].

## Conclusions

In this work, we successfully developed a simple but effective microfluidic device to investigate the role of EGF, GM6001 in invadopodia formation in the process of lung cancer cell invasion in an in vivo-like 3D microenvironment. We observed that EGF promoted invadopodia formation in 3D matrix effectively and this induction of invadopodia by EGF could be inhibited greatly by GM6001. Our results might provide new insights into the mechanism of MMP inhibitor's effect on cell invasion and support that MMP inhibitor might be a powerful candidate against invadopodia target anti-invasion therapy. This microfluidic system would be significant in exploring invadopodia formation and investigating therapeutics in a biologically relevant context. The established culture model should be suitable for analysis of the invasion mechanism and discovering anti-invasion drugs in a well defined 3D environment.
